# Percutaneous electrical nerve field stimulation compared to standard medical therapy in adolescents with functional abdominal pain disorders

**DOI:** 10.3389/fpain.2023.1251932

**Published:** 2023-09-19

**Authors:** Neha R. Santucci, Rashmi Sahay, Khalil I. El-Chammas, Kahleb Graham, Mikaela Wheatley, Madeleine Vandenbrink, Jennifer Hardy, Lin Fei

**Affiliations:** ^1^ Gastroenterology, Cincinnati Children’s Hospital Medical Center, Cincinnati, OH, United States; ^2^ Pediatrics, University of Cincinnati, Cincinnati, OH, United States; ^3^ Biostatistics, Cincinnati Children’s Hospital Medical Center, Cincinnati, OH, United States

**Keywords:** neurostimulation, amitriptyline, cyproheptadine, pediatrics, chronic abdominal pain

## Abstract

**Introduction:**

Standard medical therapy (SMT) in children with functional abdominal pain disorders (FAPD) includes cyproheptadine and amitriptyline. While percutaneous electrical nerve field stimulation (PENFS) has shown benefit, no study has compared outcomes of PENFS to SMT. We aimed to examine changes in abdominal pain, nausea and disability before and after treatment and compare outcomes between treatments.

**Methods:**

The records of FAPD patients ages 11–21 years, treated with 4 weeks of PENFS, cyproheptadine or amitriptyline were reviewed. Outcomes were evaluated using validated questionnaires [Abdominal Pain Index (API), Nausea Severity Scale (NSS), and the Functional Disability Inventory (FDI)] at baseline and follow-up within 3 months (FU).

**Result:**

Of 101 patients, 48% received PENFS, 31% cyproheptadine and 21% received amitriptyline. Median ages were 17 (15–19), 16 (15–18) and 15 (11–16) years respectively and the majority were females (75%, 90% and 52% respectively). In the PENFS group, API (*p* = 0.001), NSS (*p* = 0.059) and FDI (*p* = 0.048) were significantly lower at FU. API (*p* = 0.034) but not NSS and FDI (*p* > 0.05) decreased significantly at FU in the amitriptyline group. API, NSS and FDI did not change significantly with cyproheptadine at FU (*p* > 0.05). FU API scores were lower in PENFS vs. cyproheptadine (*p* = 0.04) but not vs. amitriptyline (*p* = 0.64). The FDI scores were significantly lower in the amitriptyline vs. cyproheptadine group (*p* = 0.03).

**Conclusion:**

Therapy with PENFS showed improvements in abdominal pain, nausea and disability while amitriptyline showed improvements in abdominal pain within 3 months of treatment. PENFS was more effective than cyproheptadine in improving abdominal pain. Amitriptyline improved disability scores more than cyproheptadine and showed promise for treatment. PENFS may be a good non-pharmacologic alternative for FAPD.

## Introduction

Functional abdominal pain disorders (FAPD), namely irritable bowel syndrome (IBS), functional dyspepsia (FD), abdominal migraine, and functional abdominal pain—not otherwise specified (FAP-NOS) have increased disability and an extensive health care cost burden (1–3). Chronic nausea and abdominal pain are debilitating symptoms that often co-exist in children with FAPD and often associated with high physical and psychosocial distress. Standard medical therapy (SMT) in children with FAPD have traditionally been sub-optimal and frequently includes off-label medications such as cyproheptadine and amitriptyline (4–6).

There has been a recent shift towards non-pharmacologic management, particularly in children, where chronic medication use can be problematic. Percutaneous electrical nerve field stimulation (PENFS) is an emerging minimally invasive approach to treat patients with chronic abdominal pain. It modulates central pain pathways through stimulation of the auricular branches of cranial nerves after just 4-weeks of treatment, with sustained efficacy (7). Several pediatric studies have demonstrated the benefits of PENFS in children with FAPD (7–9). However, no study has compared outcomes of PENFS to SMT. We aimed to compare improvements in abdominal pain, nausea and disability using validated measures between these treatment options. We hypothesized greater improvements in abdominal pain, nausea and disability with use of PENFS compared with SMT.

## Methods

After obtaining institutional review board approval, the electronic medical records of patients ages 11–21 years who met the Rome 4 criteria for a FAPD and had been treated with 4 weeks of PENFS, cyproheptadine or amitriptyline between January 2019 and December 2021 were retrospectively reviewed. Patients with organic gastrointestinal conditions known to cause abdominal pain were excluded. Demographic data and medical history were obtained from the medical record. Outcomes were evaluated using validated questionnaires that were prospectively collected routinely as part of clinical care at baseline and follow-up visit within 3 months (FU). These included:

Abdominal Pain Index (API): The API is validated for children to determine the frequency, severity, intensity and duration of abdominal pain over 2 weeks (10). We used a modified version with the duration over one week. A composite score was obtained summing individual items.
Nausea Severity Scale (NSS): The NSS, is a validated tool to determine the number of days, daily episodes and duration and intensity of nausea over 2 weeks (11). This measure normally assesses nausea severity over 2 weeks. We used a modified version with the duration over one week.
Functional Disability Inventory (FDI): The FDI is a 15 item validated instrument to measure daily physical and psychosocial functioning (12). It determines the degree of impairment caused by patients' symptoms. This measure normally assesses functioning over 2 weeks. While this measure has not been validated for shorter periods, we used a modified version with the duration over one week.

### Statistical analysis

Categorical variables were presented as frequency counts and percentages while continuous data were presented as mean (95% CI) or median (IQR). Outcomes were assessed for each group using Chi Square test. Outcomes were evaluated from baseline to FU in each group, as a three-way comparison between groups as well as pairwise comparisons between groups.

Outcomes were compared between groups at 3 months via linear mixed modeling with subject as random effect. Baseline demographic differences between groups were accounted for during linear mixed modeling. Results were presented as Least Squares Mean (LS Mean) and 95% confidence interval. All analyses were conducted as two-sided test with *p* ≤ 0.05 to be statistically significant using SAS version 9.4 (SAS Institute Inc., Cary, NC, USA).

## Results

### Demographic data

Demographic details are presented in Table 1. Of the 101 patients, 35% had FD, 35% IBS and 30% had FAP-NOS. Of these, 49 (48%) were treated with PENFS, 31 (31%) with cyproheptadine and 21 (21%) with amitriptyline. Doses for medications ranged from 10 to 50 mg nightly for amitriptyline and 2–4 mg daily to TID for cyproheptadine. In the PENFS group, 29 (59%) patients had been on medications but failed treatment and hence, received PENFS. These patients remained on a stable medication dose for the duration of treatment with PENFS. Median (IQR) ages in these groups were 17 years (15–19), 16 years (15–18) and 15 years (11–16) respectively. In all three groups, the majority were females (75%, 90% and 52% respectively) and Caucasian (98%, 87% and 95% respectively). Age and gender differed at baseline (*p* = 0.02 and *p* = 0.008 respectively) but no significant differences were noted in racial distribution between the groups.

**Table 1:** Demographic characteristics.

	PENFS	Cyproheptadine	Amitriptyline	*p*-value
	(*n* = 49)	(*n* = 31)	(*n* = 21)	
Age (year)^a^	17 (15–19)	16 (15–18)	15 (11–16)	0.02
Sex				0.008
Female	37 (75.5)	28 (90.3)	11 (52.4)	
Male	12 (24.5)	3 (9.7)	10 (47.6)	
Race				0.128
White	48 (98)	27 (97.1)	20 (95.2)	
Black	1 (2)	1 (3.2)	0 (0)	
Others	0 (0)	3 (9.7)	1 (4.8)	

All values are counts (%) unless specified.

PENFS, percutaneous electrical nerve field stimulation.

^a^ Data as median and IQR.

### Changes in measures in each group

Table 2 presents changes measures in each group. In the PENFS group, API (*p* = 0.001), NSS (*p* = 0.059) and FDI (*p* = 0.048) were significantly lower at 3-month FU compared with baseline. API scores decreased significantly at FU in the amitriptyline group (*p* = 0.034). However, NSS and FDI scores did not change significantly at FU in the amitriptyline group. All scores decreased but were not significant in the cyproheptadine group. Examining each outcome longitudinally, the API and NSS scores were lowest in the PENFS group. The FDI scores, however, were lowest in the amitriptyline group.

**Table 2:** Changes in measures in each group.

Treatment	Measure	Visit	LS means (LCL, UCL)	Diff LS means (LCL, UCL)^a^	*p*-value
PENFS	API	Baseline	2.776 (2.398, 3.153)		
		3 mFU	2.006 (1.512, 2.499)	−0.77 (−1.169, −0.371)	0.001
	NSS	Baseline	2.45 (2.039, 2.861)		
		3 mFU	1.738 (1.01, 2.466)	−0.712 (−1.456, 0.032)	0.059
	FDI	Baseline	20.244 (16.09, 24.399)		
		3 mFU	14.382 (8.215, 20.55)	−5.862 (−11.652, −0.073)	0.048
Cypro-heptadine	API	Baseline	3.555 (2.77, 4.34)		
		3 mFU	3.252 (2.456, 4.049)	−0.303 (−1.022, 0.416)	0.377
	NSS	Baseline	2.603 (2.026, 3.181)		
		3 mFU	2.054 (1.463, 2.645)	−0.550 (−1.259, 0.160)	0.117
	FDI	Baseline	23.785 (19.161, 28.408)		
		3 mFU	20.604 (15.161, 26.047)	−3.181 (−8.053, 1.691)	0.185
Amitriptyline	API	Baseline	3.113 (2.045, 4.182)		
		3 mFU	2.3 (1.186,3.413)	−0.814 (−1.553, −0.074)	0.034
	NSS	Baseline	2.007 (1.192, 2.822)		
		3 mFU	1.445 (0.579, 2.311)	−0.562 (−1.262, 0.138)	0.101
	FDI	Baseline	15.944 (8.352, 23.537)		
		3 mFU	11.709 (2.597, 20.82)	−4.236 (−12.195, 3.723)	0.259

Examined using Chi square test.

PENFS, percutaneous electrical nerve field stimulation; API, abdominal pain index; NSS, nausea severity scale; FDI, functional disability inventory; LS, least square; LCL, lower control limit; UCL, upper control limit.

^a^ negative values indicate reduction in outcome scores from baseline to 3month Follow Up visit.

### Comparison of outcomes between groups

**Figure 1:**
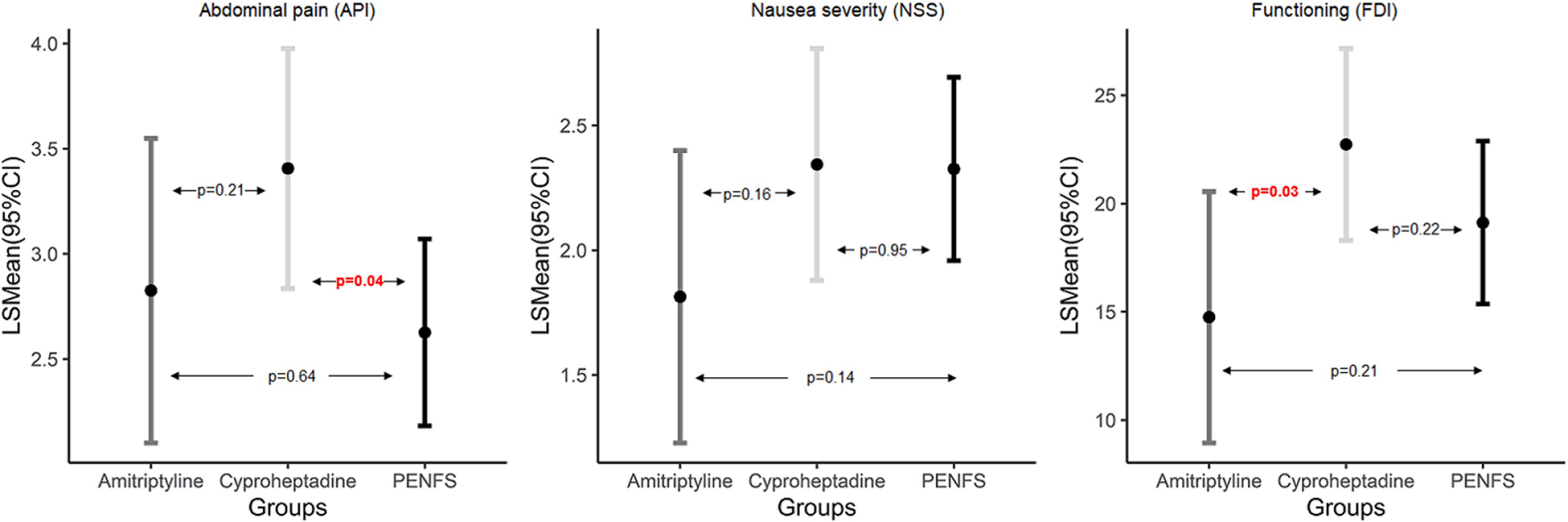
Pairwise comparison of outcomes between PENFS and SMT examined using linear mixed modeling; data presented as Least Square (LS) means and 95% Confidence Interval (CI) and *p*-values indicate pairwise group difference; PENFS, percutaneous electrical nerve field stimulation; SMT, standard medical therapy, API, abdominal pain index; NSS, nausea severity scale; FDI, functional disability inventory, *n = 101 patients.*

Table 3 represents a three-way comparison of outcomes between groups. Changes in abdominal pain and nausea were not significant between the three groups (*p* > 0.05). There was a trend for decrease in FDI in the amitriptyline group compared with both PENFS and cyproheptadine (*p* = 0.097). Figure 1 denotes pairwise comparison of outcomes between groups. API scores were significantly lower in PENFS vs. cyproheptadine (*p* = 0.04) but not between PENFS and amitriptyline (*p* = 0.64). The NSS scores did not differ between the groups (*p* > 0.05). The FDI scores were only lower in the amitriptyline vs. cyproheptadine group (*p* = 0.03).

**Table 3:** Comparison of outcomes between treatment groups.

	Groups	LS mean (95% CI)	*p*-value
API	Amitriptyline	2.825 (2.101–3.549)	0.103
	Cyproheptadine	3.406 (2.835–3.976)	
	PENFS	2.626 (2.182–3.071)	
NSS	Amitriptyline	1.813 (1.227, 2.400)	0.283
	Cyproheptadine	2.344 (1.879, 2.809)	
	PENFS	2.326 (1.958, 2.694)	
FDI	Amitriptyline	14.751 (8.944–20.557)	0.097
	Cyproheptadine	22.732 (18.304–27.159)	
	PENFS	19.12 (15.359–22.882)	

Examined using linear mixed modeling.

LS, least square; API, abdominal pain index; NSS, nausea severity scale; FDI, functional disability inventory.

## Discussion

This is the first study to compare outcomes of PENFS with standard medical therapy (amitriptyline or cyproheptadine) in adolescents with FAPD. Compared to pre-treatment,we found that PENFS significantly improved both abdominal pain and disability, with a trend for improvement in nausea, while amitriptyline significantly improved abdominal pain. PENFS was more effective than cyproheptadine in improving abdominal pain scores but did not differ from amitriptyline. Amitriptyline improved disability scores more than cyproheptadine.

Multiple biopsychosocial factors affect outcomes in FAPD. In this study, we chose to measure changes not just in abdominal pain, but also disability and nausea. This is important because FAPD are heterogenous conditions that include comorbidities once not considered as important (13). In fact, we know now that patients with co-existing nausea, for example, have worse disability (14, 15). This can result in decreased functioning which further worsens GI symptoms. Also, studies in patients with chronic pain have demonstrated the importance of functioning in terms of improving long-term outcomes, despite the presence of pain (16, 17). Thus, we chose to measure changes in disability, abdominal pain and nausea with different treatment strategies and compare outcomes.

There is a paucity of high-quality randomized placebo-controlled trials of standard medical therapy in FAPD. Several systematic reviews and meta-analysis have shown low quality of evidence to support routine use of medical therapy for FAPD and functional nausea (5, 18–20). Amitriptyline, a tricyclic antidepressant (TCA), has been commonly used in children with FAPD for years despite questionable efficacy data (21–23). The underlying mechanism for the anti-nociceptive effects of amitriptyline is not well known, but like many other drugs used to treat FAPDs, it does have strong anticholinergic properties (24). Usual doses to treat abdominal pain include 10–50 mg daily at bedtime. In addition to abdominal pain, it is commonly used to treat headaches and improve sleep at similar doses. It may also improve anxiety even at the lower doses (24). In a multicenter randomized-controlled trial including 83 children ages with FAPD, amitriptyline showed 63% improvement in pain relief and sense of overall improvement (21). However, this did not differ from placebo (58% improvement in the placebo group, *p* = 0.63). Interestingly, while amitriptyline was not better than placebo in improving pain scores in that study, it was better for reducing anxiety scores than placebo, even at the lower doses (*p* < 0.05). In contrast, a smaller double blind RCT (22) of adolescents 12–18 years with IBS (*n* = 33) showed overall improvement and reductions in abdominal pain as well as diarrhea in the amitriptyline group compared to placebo (*p* < 0.05 for each). In another study, 61% of children with FAP receiving TCA demonstrated decreased pain or improved daily functioning (23). However, this response was lesser compared to SSRIs (61% vs. 75% improvement, *p* = 0.003). TCAs, like many other antidepressants, have an FDA black box warning and can be associated with side effects such drowsiness, constipation, and worsened mood including suicidal ideation (25). Abruptly stopping the drug could lead to sleep disturbances and nightmares and overdose can be lethal (26). It is usually imperative to ask about a family history of sudden cardiac deaths suggestive of familial arrythmias prior to starting amitriptyline. While TCAs are associated with a higher risk of cardiac arrhythmias (27), studies have shown no higher risk for QTc interval prolongation in children (28, 29). It should also be noted that the long-term effects of these medications on the developing brain are unknown, and more studies are needed to determine the effects on memory and cognition in children. In our study, we noted significant improvements in abdominal pain with the use of amitriptyline in children with FAPD and the doses that were used were equivalent to what is routinely used in clinical practice and used in other studies (24, 25). However, compared to baseline, it did not significantly improve nausea or disability at 3 months post treatment. Nonetheless, changes in disability were better when compared to cyproheptadine and it can be speculated that perhaps this is due to not only improvements in pain, but the anxiolytic effects as well.

Cyproheptadine is a serotonin (5HT3) receptor antagonist with antihistaminic and anticholinergic effects, commonly used to treat FAPD in children. Doses in the range of 0.2–0.6 mg/kg/day or 4 mg up once or twice daily have been used (24). It can also improve headaches and sleep disturbances. In a small Iranian double blinded randomized controlled trial of children with FAP (30), cyproheptadine was superior to placebo in improving global symptoms (*p* = 0.005), pain intensity (*p* = 0.001) and frequency (*p* = 0.002). Similarly, a retrospective review of 80 children with dyspepsia treated with cyproheptadine showed a 55% response rate and 30% adverse event rate (31). In contrast, another retrospective study of 300 children with FAPD ages 1–18 years showed a 73% response rate and 32% adverse event rate with the use of cyproheptadine (32). Common adverse effects included sleepiness, weight gain and mood changes. While lower seizure threshold has been reported in animal models (33), this adverse effect has not been described in prior studies including pediatric DGBI patients. Surprisingly, cyproheptadine performed the poorest in the current study compared to PENFS and amitriptyline. One plausible explanation is tachyphylaxis to the drug which would require cycling to improve its sustained efficacy. Also, previous studies have reported benefits in children younger than 12 years (31) and it is possible that cyproheptadine may work better in younger children. However, more studies are needed to determine the most likely responders.

Auricular PENFS is the only FDA cleared treatment for IBS and associated abdominal pain conditions. It accesses central pain pathways and has been shown to modulate the limbic system in an animal model of IBS (34). More specifically, it decreases firing of neurons in the amygdala by greater than 50% (34). Studies have suggested that the amygdala is involved in the pathophysiology of IBS in adults (35, 36). In a randomized sham-controlled clinical trial in children with FAPD, decrease in worst pain and composite pain scores and improved disability and well-being were noted in patients receiving the active device vs. sham (7). A sub-analysis of just IBS patients showed significant improvements in abdominal pain and global symptoms after 4 weeks of PENFS treatment compared to sham (37). Interestingly, another study showed that those who responded to treatment were patients with vagal nerve insufficiency, suggesting a vagally mediated pathway (38). Improvements in resting as well as induced abdominal pain and nausea, sleep disturbances, pain catastrophizing, somatization, anxiety and disability are sustained at 6–12 months in adolescents with FAPD (8). PENFS also improved these outcomes at 3 weeks and 3 months in children with functional dyspepsia (9). Another proposed mechanism for PENFS includes microbiome changes after treatment, which may also reflect changes in vagal anti-inflammatory pathways (39). In a recent study, patients with IBS, post-PENFS treatment, were found to have decreased Clostridial species and long chain fatty acid microbial pathways that have been implicated in the pathophysiology of IBS (39). In that study, improvements in abdominal pain, functioning, and catastrophizing were noted as well. Similar to previous studies, our study confirms the benefits of PENFS in improving abdominal pain and disability. Unlike SMT, it demonstrated a trend for improvement in nausea. Compared to side effects associated with medications, no serious adverse events have previously been reported with PENFS therapy (40).

In our study, 59% of the patients in the PENFS group had failed prior SMT. It is possible that the PENFS group may have more severe, refractory symptoms compared to SMT groups which could have affected treatment outcomes. Similarly, the higher ratio of males to females in the PENFS group could also impact the results of our study.

It is interesting to consider how PENFS differs mechanistically from medications like amitriptyline that are considered “neuromodulators”. While the exact mechanism for the therapeutic effect of these drugs is not known, some have suggested different central pathways for PENFS and SMT. A recent study investigated connectivity differences between PENFS and standard medical therapy in adults with fibromyalgia (41). In that study, PENFS increased connectivity post-treatment from the right posterior insula to the right middle occipital gyrus, left midbrain, left anterior insula, and right lobule IX of the cerebellum that was associated with decreased pain scores. Conversely, those treated with standard medical therapy without PENFS, which included tricyclic antidepressants, were found to have decreased connectivity from the right posterior insula to the other brain regions. These changes were also associated with decreased pain scores. This was an interesting finding and suggests that PENFS may promote neuromodulation across brain areas and networks that are different from SMT.

This is the first study to compare treatment outcomes of PENFS with SMT. We had a moderate sample size in each group allowing for meaningful comparisons. We used validated, pediatric questionnaires that provided objective assessments however, the modified versions over 1 week were not validated compared to the standard questionnaires assessing symptoms over 2 weeks. Unfortunately, the study design did not allow us to evaluate baseline psychological comorbidities that could theoretically impact treatment outcomes. Similarly, the retrospective study design precluded assessment of other biopsychosocial factors that could contribute to symptoms. Also, data were assessed at 3 months, and it would have been ideal to have a longer follow-up with the entire cohort. Only a prospective head-to-head trial would allow for this longer follow-up since medications are typically discontinued if there are no benefits after proper dose adjustments. Future trials should also include prospective analysis of adverse event comparisons between PENFS and SMT.

In conclusion, PENFS improved abdominal pain and disability in adolescents with FAPD with a trend for improvement in nausea when assessed three months after treatment. Amitriptyline also improved abdominal pain compared to baseline. PENFS showed a trend for greater improvement in disability compared to SMT. PENFS was superior in decreasing abdominal pain compared with cyproheptadine while amitriptyline was superior to cyproheptadine in improving disability. These findings may help guide provider choices when considering pharmacotherapy in children with FAPD, particularly in cases involving patients that have side-effects to medications or drug interactions. Several studies now support PENFS as an effective treatment option to pharmacotherapy with a relatively safe side-effect profile.

## Data Availability

The raw data supporting the conclusions of this article will be made available by the authors, without undue reservation.
